# An Analysis of Job Satisfaction among Iranian Pharmacists through Various Job Characteristics 

**Published:** 2014

**Authors:** Mohamad Javad Foroughi Moghadam, Farzad Peiravian, Azadeh Naderi, Ali Rajabzadeh, Hamid Reza Rasekh

**Affiliations:** a *Department of Pharmacoeconomics and Pharma Management, School of Pharmacy, Shahid Beheshti University of Medical Sciences, Tehran, Iran. *; b *School of Pharmacy, Shahid Beheshti University of Medical Sciences, Tehran, Iran. *; c *Iran Management and Productivity Study Center, Tarbiat Modarres University (TMU) ,Tehran, Iran. *

**Keywords:** Pharmacist, Job satisfaction, Job characteristics, Job performance, Iran

## Abstract

Introduction: Pharmacists and pharmaceutical services are among the most important resources and programs in providing health for a society. Pharmacists as the key players in presenting health services, greatly impact on the health of a society and if they suffer low job satisfaction, their dissatisfaction may relatively threaten health in a society. This study was conducted to determine Iranian pharmacists’ job satisfaction and additionally, some causes of dissatisfaction among pharmacists have been diagnosed. Method: A job satisfaction questionnaire was developed and reliability tests were done by some experts in field of pharmacy practice. A sample of 700 pharmacists was selected among ten leading provinces of the country and questionnaires were distributed at the continuing pharmacy education conferences. Three essential factors named “Endogenous Satisfaction”, “Exogenous Satisfaction” and “Current Sense of Being Pharmacists” was considered as the main job satisfaction factors. Results and Discussion: Generally low scores of exogenous and endogenous job satisfaction were concluded among pharmacists while most of them were highly satisfied with being pharmacist. Male pharmacists were more satisfied than their female colleagues and a positive relationship between age and work experience with exogenous job satisfaction was found. Conclusion: Low levels of job satisfaction which were found among Iranian pharmacists could be considered as a deficiency of health system in Iran. Fortunately, inherent interest in the pharmacy profession found among Iranian pharmacists is an optimistic point at which policy-makers could develop their modifying policies. Health policy-makers must endeavor to take other steps to issue solutions for this current problem.

## Introduction

Providing, maintaining and developing public health are the main goals of a health system which will be obtained by integrating human resources and both national and international programs. Pharmacists and pharmaceutical services are among the most important resources and programs in the mentioned subject. This is while the efficiency of each system requires full and effective cooperation of all components and if any component does not work properly, it will have a negative impact on the whole system more than its own share. Pharmacists as one of the producer groups in the health sector and either one of the major components of any health system are not the exception to this rule (1). The pharmacy profession has expanded significantly in terms of professional service delivery; now it is known as an important profession in the multidisciplinary provision of health care (2). According to all these arguments, pharmacists should be at higher concern about their job performance and efficiency at work and job satisfaction is one of the major factors influencing job performance (3,4,5).

Numerous researches have been conducted in order to define the relationship and the impact of job satisfaction on job performance and efficiency. Job satisfaction is commonly conceptualized as an effective variable that results from an assessment of an individual’s job experiences. Locke (1976) defined job satisfaction as “a pleasurable or positive emotional state resulting from the appraisal on one’s job or job experiences” (6). Job satisfaction is conceptualized in general as one’s attitude toward his or her job. As a total of all various definitions of job satisfaction, the concept can be defined in a simple manner as the degree to which people like their jobs (7).

Robbins (1991) proved in his study that personnel dissatisfied from their job are at risk of non-healthy conditions from headache to heart diseases (8). Steers (1981) indicates that those who are satisfied with their job, complain less, live more, have better physical and mental health, learn new job tasks earlier and have less occupational accidents (9). Job dissatisfaction even causes physical and mental discomfort, family instability, lack of social cohesion and eventually political imbalance (10).

Along with all negative impacts of job dissatisfaction, a review article by Schafheutle *et al. *indicates that pharmacists’ performance is affected by four key domains. One of the domains included workplace factors like working pattern, work environment, sector of practice and external [including commercial] pressures. In the reviewed literature, job dissatisfaction shows negative impact on pharmacists’ job performance and increases dispensing errors. On the other side, one of the other domains affecting job performance is mental and physical health (chronic illness, stress, depression, alcohol, drug and other addictions, cognitive impairment) (11). As it was previously mentioned, mental and physical health are themselves injured by job dissatisfaction and dissatisfaction with work has long been associated with poor mental health (12,13).

Coming back to the field of health services, it is very important for health policy makers to concern the improved performance of health professionals such as pharmacists which are directly in contact with patients. Governments in Canada, Britain and other developed countries are increasingly recognizing the potential of a fuller integration of pharmacists in primary health care as a means of improving the public’s health and reducing drug-related iatrogenesis (14). Pharmacists as the key players in delivering health services greatly impact the health of society and if they suffer low job satisfaction, relatively will threaten the health of society. Because it is strongly admitted that unhappy and demotivated workforces have a negative impact on the delivery of healthcare services and the experience of patients in receipt of care (15). 

*Aim of the study*

In line with expansion of pharmacy schools, growth in pharmacy graduates, high expenditure on human resource training and more importantly with a look at the effective role of pharmacists in health system, this study will determine the level of job satisfaction through various aspects of pharmacy practice among Iranian pharmacists. Furthermore some satisfaction or dissatisfaction causes among pharmacists will be explored and may help policy makers to focus on these elements when aiming at the improvement of health care provision. 

## Experimental

*Questionnaire*

Because no validated questionnaire has ever been adapted to Iranian pharmacists, our research seeks to establish such a questionnaire. The job satisfaction questionnaire was developed based on expert opinions and review of similar studies especially the one in the culturally similar country: Lebanon. Salameh *et al. *considered work content, autonomy, growth/development, financial rewards, promotion, supervision, communication, coworkers, meaningfulness, workload, and work demands as factors of job satisfaction among pharmacists in Lebanon (16). 

In order to assess the content validity of the designed questionnaire, it was assessed by 36 experts which were pharmacists working in academic environments with years of experience in pharmacy practice and familiar with regulations and working conditions in pharmacy practice in Iran. These experts were asked to rate their agreement with each question regarding the following criteria of validity while score ‹5› stood for ‹completely satisfactory›, ‹4› for ‹somewhat satisfactory›, ‹3› for ‹acceptable›, ‹2› for ‹needs change› and ‹1› for ‹needs replacement›. 

• Linguistic Clarity – The item should be clear, unambiguous, and jargon-free, and attract a spontaneous response rather than requiring pondering on the part of the respondent. 

• Completeness – The item’s sentence structure should be complete and articulated in conveying the underlying concept with emphasis at the right places. 

• Relevance – The item should be relevant to the area under study and what is being measured and have the right focus. 

• Scoring – The Likert scale response options (1 to 5) and its anchors should fit the item’s sentence structure and the way it is phrased.

The content validation procedure was performed in one step. The analysis of the results was carried out to demonstrate the consensus among the experts. After primary evaluation of the questionnaire, some questions which were not considered relevant were deleted and other debatable ones were reformed. The validated questionnaire was piloted by a sample of 30 pharmacists working in community pharmacy which had been selected by quota sampling. Correlation matrices were calculated for the satisfaction items and Cronbach alpha was measured to determine if the items in the constructed questionnaire was measuring the same underlying concept (satisfaction). A Cronbach alpha score of 0.86 or greater was considered to be evidence of good reliability.

The conducted questionnaire had three parts. The first part included 13 questions about age, gender, marital status, number of children, high school and university they were graduated from and the priority of the field of pharmacy they had been selected to study. In part two, they were asked about sectors they were working in, and in part three, satisfaction about some aspects of job was questioned and quantified based on a 5-point Likert Scale in which 1 stood for ‘very low satisfaction’, 2 for ‘low satisfaction’, 3 for ‘neither satisfied nor dissatisfied’, 4 ‘high satisfaction’, and 5 for ‘very high satisfaction’.

These job characteristics were divided into two parts including endogenous and exogenous job characteristics. Endogenous job characteristics included Overall Job Satisfaction, Income, Job Security, Awareness of Regulations and Job Expectancy. On the other hand, exogenous characteristics included Job Position, Performance of Associations of Pharmacists (Unions), Intention to Work Abroad, Reimbursement from Insurance Organizations to Pharmacies, Pharmacy Supervision, Pharmacy Evaluation and Implementation of Regulations.

*Sampling*

As far as there was no information on Iranian pharmacists’ population variance neither on the probability of success neither the failure, the biggest sample size based on Morgan table which is 374 for a population of 13000 pharmacists registered in Iran was enough. For higher confidence and considering the probability of pharmacists who were not working or even have immigrated, a sample of 700 pharmacists was selected among ten leading provinces of the country based on quota sampling. Pharmacists (700) were asked to complete the self-administered questionnaire at the Continuing Medical Education (CME) courses at which pharmacists all over the country have to participate. 

The total selected number in each of ten clusters was based on the population of the province which is a predictor of pharmacists’ population. Pharmacists in CMEs were assured about the secrecy of answers and were informed about the purpose of the study and then a questionnaire based survey was completed. Finally 575 filled questionnaires were gathered in all ten provinces which show a response rate of 82 %. 

*Statistical analysis*

Data were entered into SPSS (version 16) and analyzed using descriptive statistics, t-test and regression analysis. The significance level was set at P<0.05. Data was first analyzed descriptively with modes, means, and standard deviations, for discrete and continuous variables. Frequencies and valid percentages for categorical variables were calculated. In order to determine the relationship between demographic data with job satisfaction, non-parametric tests were used. This was to avoid making assumptions about the distributions of the variables and in view of the Likert scale being ordinal data. This is consistent with the analytical approach of previous studies (17,18,19,20). Mann-Whitney and Kruskal-Wallis tests were used for this purpose. The regression and correlation analyses were used to show relationship between job characteristics and job satisfaction (21).

## Results

The demographic characteristics of 575 respondent (Response Rate ~ 82%) pharmacists derived from part one and two of questionnaires are presented below.

The respondents were between 25 to 77 years old and the average age was 42. Most respondents were between 30 to 50 years old (68%). About 55% of the participants were male and 45% were female. About 91 percent of participants were married and number of their children followed a mode of 2. More than half of the respondents mentioned that even before they start to study at university their favorite field had been pharmacy and for 39% of respondents, pharmacy field had been the first choice of study in the national university admission test.

About 87% of respondents were working in pharmacies and 13% in other sectors like pharmaceutical manufacturing, import, export and distribution companies, research laboratories, governmental sectors and health insurance organizations. The data also indicated that 54 percent of respondents had their own private pharmacy and the others were employed by private or governmental organizations. Iranian national regulation do not allow establishment of chain pharmacies. In addition, same regulation would permit only pharmacists to establish and own a pharmacy in Iran.

The mean value of job satisfaction based on data entered in part three of questionnaires were analyzed and t-test was used for measuring the significance of difference between mean value of every scored job characteristic and the test value of 3 as medium satisfaction. Every mean more than 3 means high satisfaction and vice versa every mean value less than 3 means low satisfaction level.

As mentioned, job characteristics were categorized to endogenous and exogenous. The two parameters named *endogenous job satisfaction *and *exogenous job satisfaction *were calculated as the mean of all job characteristics. After all questions, pharmacists were asked to answer one key question to mention their *current sense *of being a pharmacist and this will be a dependent variable in regression analysis between all job characteristics. In this question, respondents were asked to mention their overall mental perception about their career and this will show their real inherent satisfaction. 

Note that all mean values were significantly different from mean 3 just in case of intention to work outside the country and level of awareness in regulations (p<0.05). The accessed information through statistical analyses shows significantly low job satisfaction through endogenous and exogenous satisfaction but respondents drive nearly high satisfaction from their career (*Current Sense*).

For more briefing on some questions the structure of reimbursement is as follows. Patients pay about 30% (different in various insurance structures) of the prescription cost as the co-insurance directly to the pharmacies and pharmacies receive the other 70% from the insurance organization. Most of the time, this reimbursement is very late and bureaucratic and makes pharmacy owners unhappy and dissatisfied with those organizations. In the job expectancy question, pharmacists were asked how much optimistic they were about the future of their job. (Table 1).

**Table 1:** Job satisfaction analysis through various job characteristics.

	**Mean Satisfaction**	**SD**	**t-test results**						
			**N**	**df**	**t**		**Sig (2-tailed)**	
Job Satisfaction “Endogenous” (Average)	2.73	0.70	549	548		-8.96		0.000	
Overall Job	3.1	0.59	570	569		2.40		0.017	
Income	2.62	0.81	267	566		-1.12		0.000	
Job Security	2.38	0.94	569	568		-1.56		0.000	
Regulations Awareness	3.04	0.86	563	562		1.03		0.302	
Job Expectancy	2.48	0.99	559	558		-1.24		0.000	
Job Satisfaction “Exogenous” (Average)	2.49	0.66	505	504		-1.74		0.000	
Job Position	2.73	0.97	569	568		-6.50		0.000	
Association of Pharmacists (Unions)	2.35	1.36	563	562		-1.12		0.000	
Intention to Work Abroad	3.01	1.33	561	560		0.13		0.899	
Reimbursement System	1.67	0.78	552	551		-3.97		0.000	
Pharmacy Supervision	2.64	1.07	565	564		-7.90		0.000	
Pharmacy Evaluation	2.49	0.89	544	543		-1.34		0.000	
Regulations Implementation	2.54	0.87	549	548		-1.22		0.000	
Current Sense	3.51	1.01	554	553		11.86		0.000	

Mann-Whitney test was done for analyzing difference between two groups of respondents demographically –*e.g*. men and women- in endogenous job satisfaction, exogenous job satisfaction, and the key characteristic which is *current sense*. As it is seen in Table 2, men showed significantly higher job satisfaction than women through endogenous job characteristics but in case of exogenous job characteristics and even *current sense *there is no significant difference between men and women’s ideas.

Those whose favorite field had been pharmacy mentioned significantly higher job satisfaction and higher inherent satisfaction with their career. Pharmacy owner and employees showed no significant different in case of job satisfaction, but the half who owned a pharmacy were more satisfied with their income (significance level: 0.026) (Table 2).

**Table 2:** Mann-Whitney test results

**Grouping Variable**		**Test Variables Mean Rank**		
		**Endogenous Satisfaction**	**Exogenous Satisfaction**	**Current Sense**
**Gender**	Male	282.97	258.43	266.51
	Female	257.70	240.15	284.39
	Sig. (2-tailed)	0.036*	0.120	0.148
**Marital Status**	Married	269.76	251.60	275.68
	Single	256.49	214.96	242.38
	Sig. (2-tailed)	0.515	0.079	0.110
**Work Field**	Pharmacy	269.99	274.05	273.99
	Non-pharmacy	287.05	274.28	278.28
	Sig. (2-tailed)	0.360	0.146	0.821
**Favorite Field**	Pharmacy	273.29	252.82	283.36
	Non-pharmacy	240.76	226.77	236.97
	Sig. (2-tailed)	0.006*	0.023*	0.000*
**Pharmacy Ownership**	Yes	261.90	248.76	269.78
	No	273.09	243.64	270.26
	Sig. (2-tailed)	0.350	0.660	0.969

* Significant correlations are shown with stars.

For analyzing the difference between more than two groups demographically in response to job satisfaction items, Kruskal-Wallis test was used. As it is shown in Table 3, pharmacists older than 50 years old and with more than 30 years of work experience, mentioned higher job satisfaction through exogenous job characteristics and this difference was significant. Pharmacists who were working in capital city mentioned lowest job satisfaction at significance level of 0.045 (Table 3).

**Table 3:** Kruskal-Wallis test results

**Grouping Variable**		**Test Variables Mean Rank**			
		**Endogenous Satisfaction**	**Exogenous Satisfaction**		**Current Sense**
Age Categories (Years)	≤30	286.87	227.15		285.61
	31-40	250.02	221.03		252.38
	41-50	272.25	252.16		275.30
	51-60	245.69	294.23		257.21
	>60	241.59	272.91		246.64
	Sig. (2-tailed)	0.164	0.002*		0.298
Number of Children	0	263.96		197.26	249.93
	1	259.57	238.17		272.09
	2	251.61	241.80		247.21
	3	264.75	287.08		292.73
	Sig. (2-tailed)	0.835	0.000*		0.057
Working location (Based on Geographical zone of c Working)	Capital	264.64		223.99	265.27
	Middle	266.52	226.58		247.58
	Middle-Northern	326.34	293.48		337.91
	Middle-Southern	272.72	277.44		274.48
	North-Eastern	256.85	276.79		271.59
	North-Western	269.05	278.27		277.76
	Northern	267.91	234.79		298.56
	South-Eastern	310.45	253.89		321.92
	Southern	286.09	254.90		238.43
	Western	290.75	237.85		279.79
	Sig. (2-tailed)	0.471	0.045*		0.085
Working Experience (Years)	40-50	257.00		310.68	280.87
	30-39	259.30	276.46		250.74
	20-29	251.66	267.68		259.14
	10-19	271.79	242.57		278.59
	1-9	267.27	228.63		263.29
	Sig. (2-tailed)	0.826	0.043*		0.623
Priority of Pharmacy Field	1	266.61		245.92	262.50
	2	255.38	244.26		264.58
	3-100	249.60	224.83		252.19
	Sig. (2-tailed)	0.464	0.257		0.681

* Significant correlations are shown with stars

In next step for analyzing weight of job satisfaction parameters, Friedman test was done and as it is detailed in Table 4, maximum scores were given to satisfaction with job (overall job satisfaction) and the least scores were given to insurance system performance.

**Table 4:** Friedman Ranking Test Results

			Item	**Mean Rank**
N= 498 Chi-Square = 9.619 df= 11 Sig.= 0.000	Job Satisfaction Characteristics	1	Overall job satisfaction	8.47	
		2	Regulations awareness	8.17	
		3	Intention to work abroad	7.56	
		4	Position among medical groups	7.10	
		5	Income level	6.71	
		6	Pharmacy supervision	6.71	
		7	Regulations implementation	6.40
		8	Pharmacy evaluation	6.26
		9	Job expectancy	6.06
		10	Job security	5.80
		11	Association of Pharmacists (Unions)	5.45
		12	Reimbursement System	3.31

To examine the relationship between the job characteristics and satisfaction, multiple regression models were examined. Regression analysis of job characteristics with dependent variable-*current sense-*shows significant linear relationship between “*Overall Job Satisfaction*”, “*Job Position*”, “*Job Expectancy*”, “*Job Security*”, “*Implementation of Regulations*” and “*Reimbursement System *”. Other characteristics did not have significant impact on satisfaction. Based on coefficients from regression analysis, satisfaction is a function of mentioned factors. More detailed results are shown in Table 5.

**Table 5:** Regression test results

	**Predictor (Independent Variable)**	**Beta Coefficient**	**t**	**Sig.**
R= 0.676 R Square= 0.457 Adjusted R^2 ^= 0.451 df= 482 F= 67.744 Sig. = 0.000	(Constant)	0.995	6.841	0.000
	Overall job satisfaction	0.414	9.996	0.000
	Job Position	0.161	3.819	0.000
	Job Expectancy	0.106	2.391	0.017
	Job Security	0.135	3.170	0.002
	Regulations Implementation	0.087	2.342	0.020
	Insurance System	-0.073	-2.030	0.043

Dependent Variable: Current Sense

## Discussion

This study was aimed to analyze the level of job satisfaction through various job characteristics compared with various locational and work settings of pharmacists and across demographics of Iranian pharmacists. According to what Salameh *et al. *(2007) noted in their article, low states of job satisfaction were also found in a developing country like Iran (16). Findings suggest that although community pharmacists enjoy aspects of their new roles, their work environment has become increasingly stressful, resulting in decreased job satisfaction. Additionally, it was previously found that increasing workloads can be resulted in decreased health and well-being (22).

Findings show no significant difference between pharmacists owning pharmacy and those not owning. This is not in consistence with the hypothesis mentioned by Lin *et al. *(2007), stating that the closer to ownership, the higher enriched job and its satisfaction (23). Pharmacy owners however drive significantly high satisfaction from their income. Also Willett (1998) noted overall job satisfaction was greatest for pharmacy owners and lowest for locum pharmacists (24) but the same job satisfaction as for pharmacists who were employed may be as a result of insurance reimbursement and governmental supervisions which is implemented strictly on pharmacy owners in Iran. 

In case of inter-gender differences, higher job satisfaction for male pharmacists through endogenous job characteristics like job expectancy, income, job security and current sense was found but no significant difference between male and female pharmacists was found in other job characteristics. This could be explained by higher stress level in female pharmacists as explained by McCann *et al. *(2009) (25). On the other hand Seston *et al*. (2009) found female pharmacists more satisfied with their job and refers to generally findings of higher job satisfaction among females (26). It was reconfirmed in this study that age does have positive impact on job satisfaction (27). As shown in Table 4, pharmacists older than 50 years and with more than 30 years’ work experience, mentioned higher job satisfaction through exogenous job characteristics and this difference was significant while dissatisfaction was most observed in younger pharmacists which is consistent with other studies (28).

In this study it was clarified that inherent interest in work does have positive impact on job satisfaction. For more explanation, pharmacists who their favorite field was pharmacy, were significantly more satisfied with their job. However more studies should be done to prove this hypothesis. 

Pharmacists working in capital city mentioned less satisfaction with exogenous characteristics. This could be explained by harder living conditions and lower income to expenditure ratio in capital city. More over in small cities pharmacists have better-organized associations and proper relationship with their coworkers. Considering the aforementioned issue, it could be suggested that professional associations like Iranian Association of Pharmacists can empower human resource in pharmacy service through doing their duties and responsibilities more seriously.

Another suggestion for arising pharmacists’ job satisfaction based on lowest ranks in Friedman test is to revise the insurance system performance in reimbursement to pharmacies. They should be reimbursed as soon as possible after forwarding the prescriptions to national and private insurance organizations so that they will be more satisfied with their income either with the job security. Furthermore increasing income will certainly increase job satisfaction based on the reviewed literature (29,30,31,32).

Generally, it could be clearly seen that overall job satisfaction as a key indicator of endogenous job characteristics and so *“current sense” *which indicates the inherent and substantial satisfaction with the profession of pharmacy are at high level so it is concluded that a vast majority of Iranian pharmacists are satisfied with being a pharmacist. Furthermore, this factor which targets endogenous satisfaction to a job can further be improved upon improvement of factors such as income, unions, and insurance. Policy-makers can perform better based on this positive perception. This can be done by providing further reinforcing activities such as annual awards and capacity building in training and educating professionals. For more explanation, if changeable characteristics like union relationships, insurance reimbursements, income and *etc*. are modified, it could effectively increase this possessive sense to the profession. Similarly, this idea could be approved through participants’ notes at the end of questionnaires. As a result of increasing this positive substantial interest in this profession, significant improvements in health of society will be gained.

There were some possible limitations in this study like non-objective comprehension of questions in the survey, just like all questionnaire-based surveys. For example mentioned satisfaction levels reflect subjective opinions of well-being and dissatisfaction might be a result of non-job related stressful factors, such as familial or environmental situations.

## Conclusion

Considering the impact of pharmacists as key players in providing health and wellbeing to the society, it should be noted that low levels of job satisfaction found among Iranian pharmacists could be considered as a deficiency of health system in Iran. Based on these findings and some offered solutions like modifying exogenous factors, policy makers must pay special attention to modify this deficiency. On the other hand, inherent interest in this career can positively impact job satisfaction. It could be used as a powerful leverage by health policy makers for their mission of improving health and elevating level of wellbeing. 

As well as already mentioned relationship between demographic characteristics and job satisfaction, inter-gender differences of job satisfaction in the field of pharmacy practice remains in doubt or it may be different based on the nationality and culture.
